# CCCH protein-PvCCCH69 acted as a repressor for leaf senescence through suppressing ABA-signaling pathway

**DOI:** 10.1038/s41438-021-00604-0

**Published:** 2021-07-07

**Authors:** Zheni Xie, Guohui Yu, Shanshan Lei, Chenchen Zhang, Bin Xu, Bingru Huang

**Affiliations:** 1 College of Agro-grassland Science, Nanjing Agricultural University, Nanjing, 210095 China; 2 Department of Plant Biology and Pathology, Rutgers the State University of New Jersey, New Brunswick, NJ 08901 USA

**Keywords:** Plant sciences, Plant biotechnology

## Abstract

CCCH is a subfamily of zinc finger proteins involved in plant growth, development, and stresses response. The function of CCCH in regulating leaf senescence, especially its roles in abscisic acid (ABA)-mediated leaf senescence is largely unknown. The objective of this study was to determine functions and mechanisms of *CCCH* gene in regulating leaf senescence in switchgrass (*Panicum virgatum*). A *CCCH* gene, *PvCCCH69* (Pv*C3H69)*, was cloned from switchgrass. Overexpressing Pv*C3H69* in rice suppressed both natural senescence with leaf aging and dark-induced leaf senescence. Endogenous ABA content, ABA biosynthesis genes (*NCED3, NCED5*, and *AAO3*), and ABA signaling-related genes (*SnRKs*, *ABI5*, and *ABF2/3/4*) exhibited significantly lower levels in senescencing leaves of *PvC3H69-*OE plants than those in WT plants. Pv*C3H69*-suppression of leaf senescence was associated with transcriptional upregulation of genes mainly involved in the light-dependent process of photosynthesis, including light-harvesting complex proteins, PSI proteins, and PSII proteins and downregulation of ABA biosynthesis and signaling genes and senescence-associated genes. *PvC3H69* could act as a repressor for leaf senescence via upregulating photosynthetic proteins and repressing ABA synthesis and ABA signaling pathways.

## Introduction

Natural or stress-induced leaf senescence adversely affects photosynthetic capacity and plant productivity^1–3^. Leaf senescence development is regulated at multiple levels, involving molecular, transcriptional, posttranscriptional, and metabolic processes^3–5^. At the transcriptional level, CCCH zinc finger proteins with three Cys and one His residues as the conserved motif that function as RNA-binding proteins and regulate RNA metabolism have been found to act as key regulators of leaf senescence in *Arabidopsis thaliana* and rice (*Oryza.Sativa*). *CCCH* genes, *AtKHZ1* and *AtKHZ2*, can accelerate leaf senescence when overexpressed in Arabidopsis^6^. *OsDOS* and *OsTZF1* were found to be negative regulators of leaf senescence in rice^7–9^. Despite the knowledge of the involvement of *CCCH* genes in leaf senescence, the upstream and downstream regulatory mechanisms of *CCCHs* controlling leaf senescence remain largely unknown.

Several *CCCH* genes have been found to interact with abscisic acid (ABA), a well-known stress hormone, in the regulation of seed germination, plant growth, and stress responses. A *CCCH* gene cloned from Arabidopsis, *AtTZF1*, was reported to upregulate the expression of ABA-response genes, *RD29A* and *COR15A*, for regulating plant growth and stress responses^4^. Other *CCCH* genes in Arabidopsis, including *AtC3H49* (*AtTZF3*) and *AtC3H20* (*AtTZF2*) was inducible by ABA and also feed forward to upregulate ABA-response genes, such as *RD29B* during drought stress^10^. *AtTZF4/5/6* genes are found to be upregulated under ABA treatment and in turn enhance ABA biosynthesis and signaling by activating *NCED9* and *ABI5* during seed germination^11^. Furthermore, AtTZF4 (SOMNUS) can be directly targeted by ABI5 and ABI3 in the process of seed germination^12,13^. AtTZF5 can interact with two ABA-response proteins, MARD1 and RD21A, during drought responses in Arabidopsis^14^. In rice, *OsC3H47* can also be induced by ABA and promote drought tolerance, but the regulatory mechanism has not been reported^15^. Some CCCH proteins from other species, such as GhZFP1 in cotton and IbC3H18 in sweet potato can also interact with ABA downstream regulators^16,17^. ABA is well-known to play crucial roles in inducing or accelerating leaf senescence, involving activation of ABA biosynthesis genes (i.e., *NCED9, ABA2, AAO3*) or ABA signaling genes (i.e., *ABI5, ABF2, ABF3*)^18–22^. However, whether and how CCCH may interact with ABA regulating leaf senescence are not well understood.

Switchgrass is a perennial warm-season bunchgrass producing feathery delicate flowers from July to September, which is widely used as ornamental grasses in warm climate regions. We have previously cloned a *CCCH* gene in switchgrass, *PvC3H69*, which is phylogenetically homologous to *OsTZF1*; the expression of this gene can be induced by ABA whereas its overexpression resulted in delaying leaf senescence in rice^8,9,23,24^. Here, we hypothesized that PvC3H69 may serve as a repressor for leaf senescence, which could be activated by ABA accumulation at the onset of leaf senescence. The objectives of this study were to characterize the function of *PvC3H69* in leaf senescence and determine whether *PvC3H69* participate ABA-mediated leaf senescence and how it may regulate the ABA-mediated leaf senescence with a goal to clarify the regulatory mechanism of *PvC3H69* and ABA signaling in leaf senescence. The regulatory mechanisms of *PvC3H69* were examined by endogenous ABA detection and exogenous application of ABA to find the main signaling pathway in plants overexpressing *PvC3H69*.

## Results

### PvC3H69 as a nuclear-localized protein without transactivation function

**Fig. 1:**
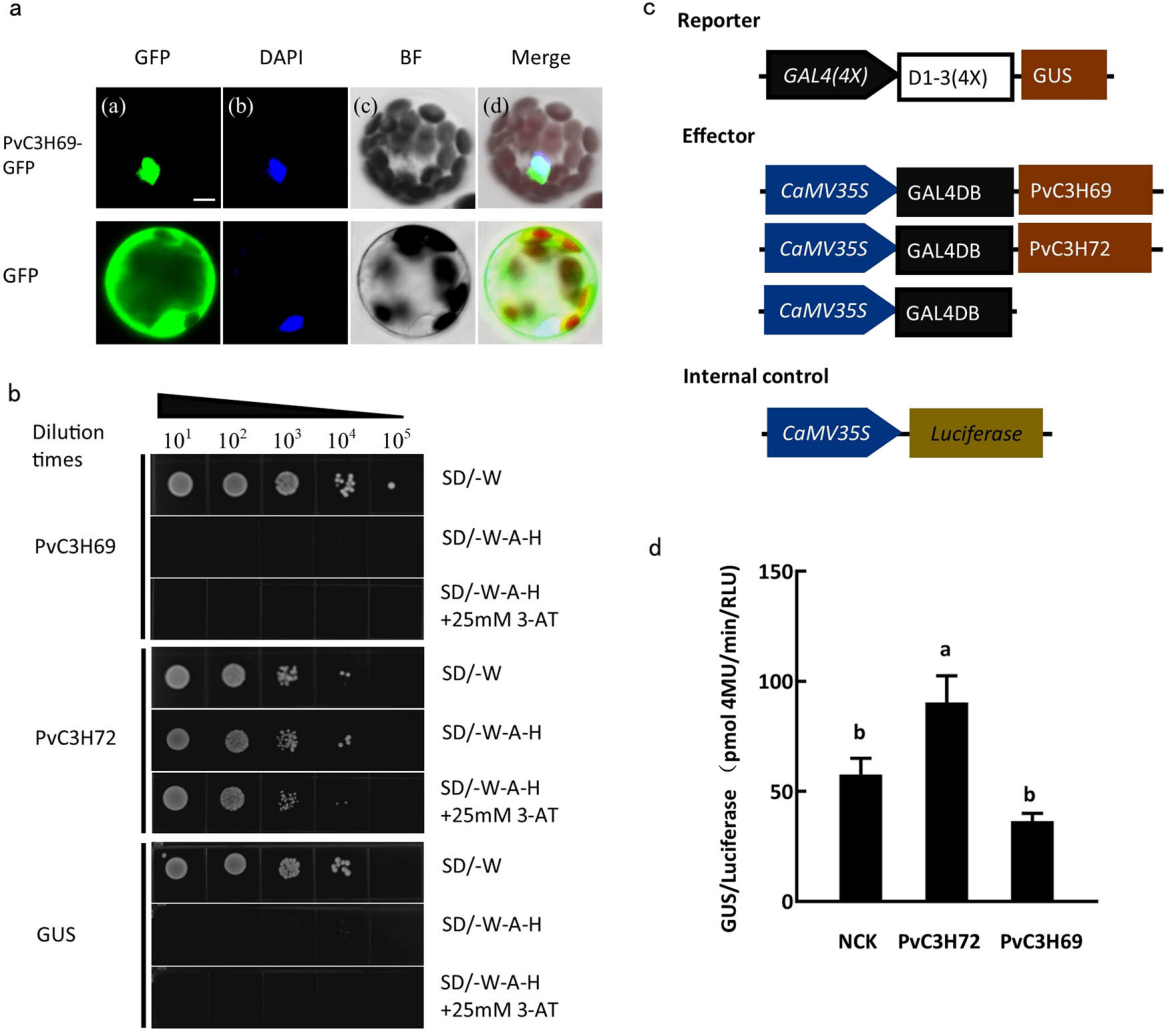
Subcellular location and transcriptional activity. **a** Subcellular location of PvC3H69. (a) PvC3H69-GFP fusion protein location, (b) DAPI, (c) BR (bright region), and (d) the merge of PvC3H69-GFP, DAPI, and BR. **b** Transcriptional activity of PvC3H69 in yeast. **c** Structural diagram of three plasmids used in transcription activity experiment. **d** Gus/Luciferase activity measurement. All the experiments were repeated three times with similar results. Letters above bars indicate significant difference at *P* < 0.05. Bar = 5 μm

The Subcellular localization of PvC3H69 was studied by fusing it with a GFP tag. From Fig. 1a, it clearly showed that GFP signal of PvC3H69-GFP was merged with the DAPI stained nuclear, while the GFP control was dispersed in nuclear and cytosol, indicating that PvC3H69 was exclusively localized in the nuclear.

To test whether PvC3H69 had transcriptional activity, we performed yeast-based transactivation assay. As shown in Fig. 1b, PvC3H69 and the negative control (GUS) fused with the GAL4 DNA-binding domain (GAL4-DB) did not activate the reporter gene in the yeast system, while the positive control of a known transcription factor, PvC3H72, had transactivity^25^. Furthermore, we carried out in planta transcriptional activity assay by transient co-expressing *35S::PvC3H69-GAL4-DB* (effector), *GAL4(4x)*::*GUS* (reporter), and *35S::LUC* (internal control) in Arabidopsis protoplasts, with *35S::PvC3H72-GAL4-DB* and *35S::GAL4-DB* as the positive and empty vector controls, respectively (Fig. 1c). The positive control of PvC3H72 activated the expression of the *GUS* reporter gene, while PvC3H69 did not activate or suppress the expression of the *GUS* reporter gene (Fig. 1d). Taken together, PvC3H69 showed no transcriptional activity in neither yeast-based nor in planta assay.

### Suppression of dark-induced leaf senescence in switchgrass and rice by overexpression of *PvC3H69*

**Fig. 2:**
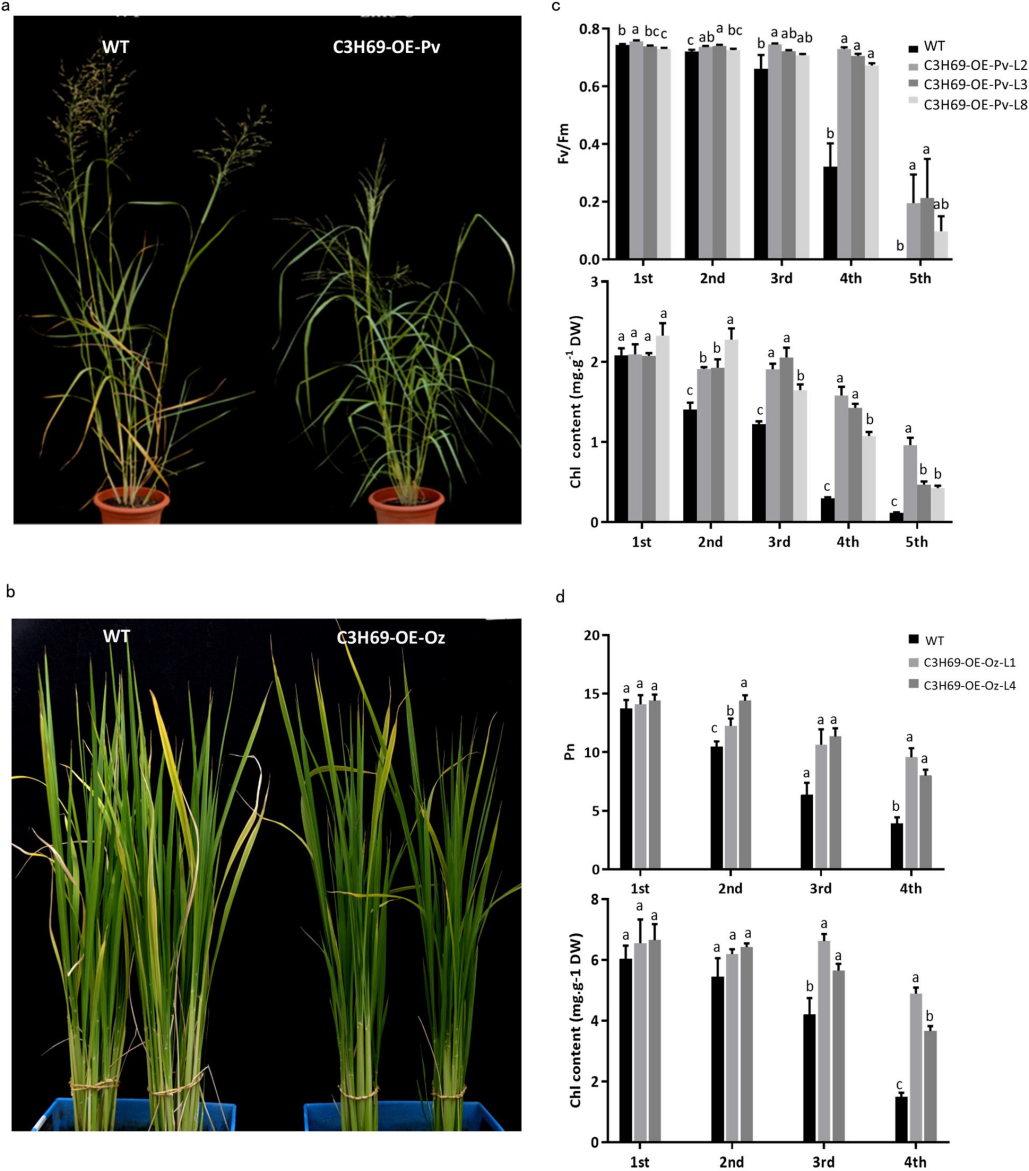
Phenotype and physiological parameters for natural leaf senescence in switchgrass and rice. **a** Natural senescence phenotype of 5-month-old switchgrass WT and transgenic lines (named as C3H69-OE-Pv). **b** Natural senescence phenotype of rice WT and transgenic lines (C3H69-OE-Oz). **c** Fv/Fm and chlorophyll content in different leaf position of a plant in switchgrass WT and transgenic lines. **d** Pn and chlorophyll content in different position of a plant in two rice C3H69-OE-Oz lines and WT. Letters above bars indicate significant difference at *P* < 0.05

**Fig. 3:**
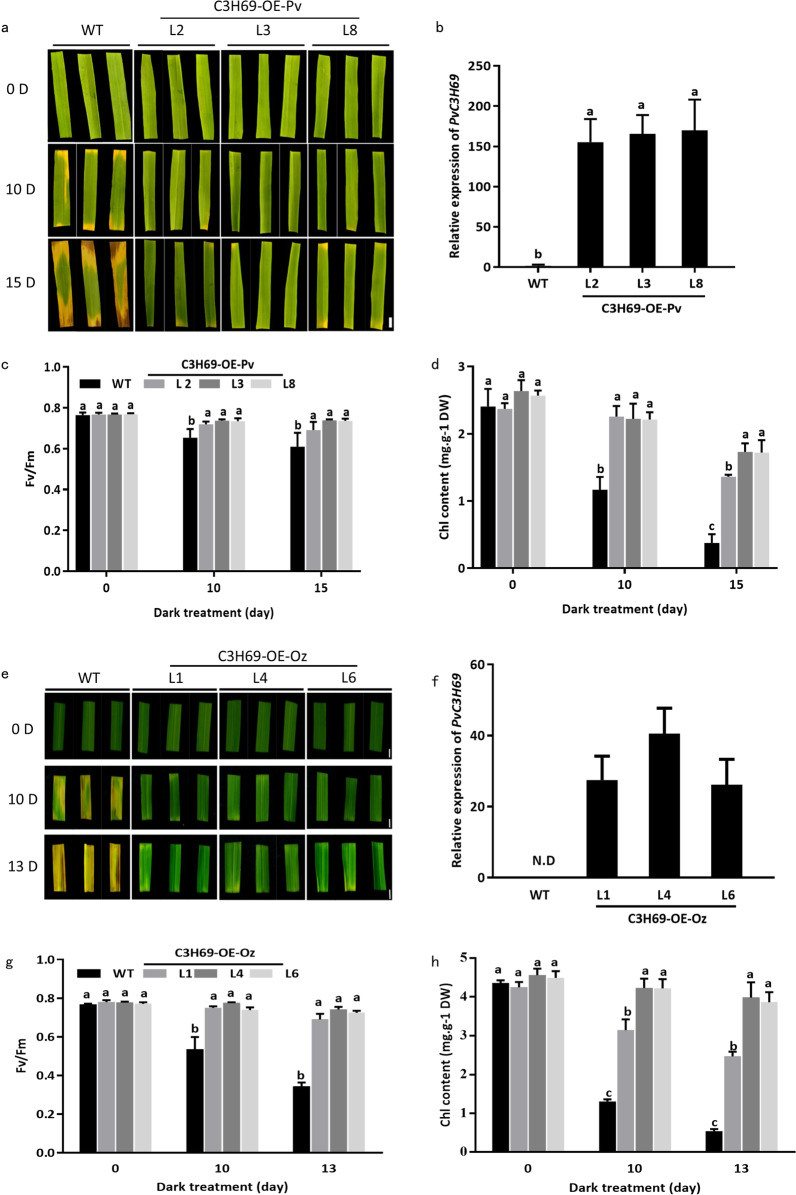
Phenotype and physiological parameters for dark-induced senescence in switchgrass and rice. **a** Detached leaves of swichgrass WT and C3H69-OE lines exposed to dark treatment. **b** Relative expression level of *PvC3H69* in switchgrass WT and C3H69-OE-Pv lines under dark treatment. **c**, **d** Fv/Fm and chlorophyll content in switchgrass WT and C3H69-OE-Pv lines under dark treatment. **e** Detached leaves of rice WT and C3H69-OE-Oz lines under dark treatment. **f** Relative expression level of *PvC3H69* in rice WT and C3H69-OE-Oz lines. **g**, **h** Fv/Fm and chlorophyll content in rice WT and C3H69-OE-Oz lines under dark treatment. Letters above bars indicate significant difference at *P* < 0.05. Bar = 0.5 cm

To understand the functions of *PvC3H69* regulating leaf senescence, we overexpressed the gene under driven of maize ubiquitin promoter in switchgrass and rice by *Agrobacterium*-mediated transformation, with transformation being confirmed by PCR and GUS staining (Fig. S1a–d). Overexpressing *PvC3H69* (abbreviated as C3H69-OE hereafter) resulted in transgenic plants of both switchgrass and rice retained more green leaves in each tiller and lower level of leaf senescence compared to their respective wild-type (WT) plants (Fig. 2a, b). Leaf photochemical efficiency (Fv/Fm) and net photosynthetic rate (Pn) of C3H69-OE lines were significantly higher than that of WT. Chlorophyll (Chl) content of the second to fourth leaves showed significantly higher levels in C3H69-OE lines than that in WT plants (Fig. 2c, d). Under dark conditions, leaves of WT plants exhibited severe leaf senescence with 90% loss of Chl content, whereas Chl content of transgenic lines maintained at 50% of the control level; Fv/Fm of transgenic lines also were significantly higher than that of WT (Fig. 3a–h). Those results demonstrated that *PvC3H69* played a positive role in suppressing dark-induced leaf senescence.

### Suppression of ABA synthesis and signaling by overexpression of *PvC3H69*

**Fig. 4:**
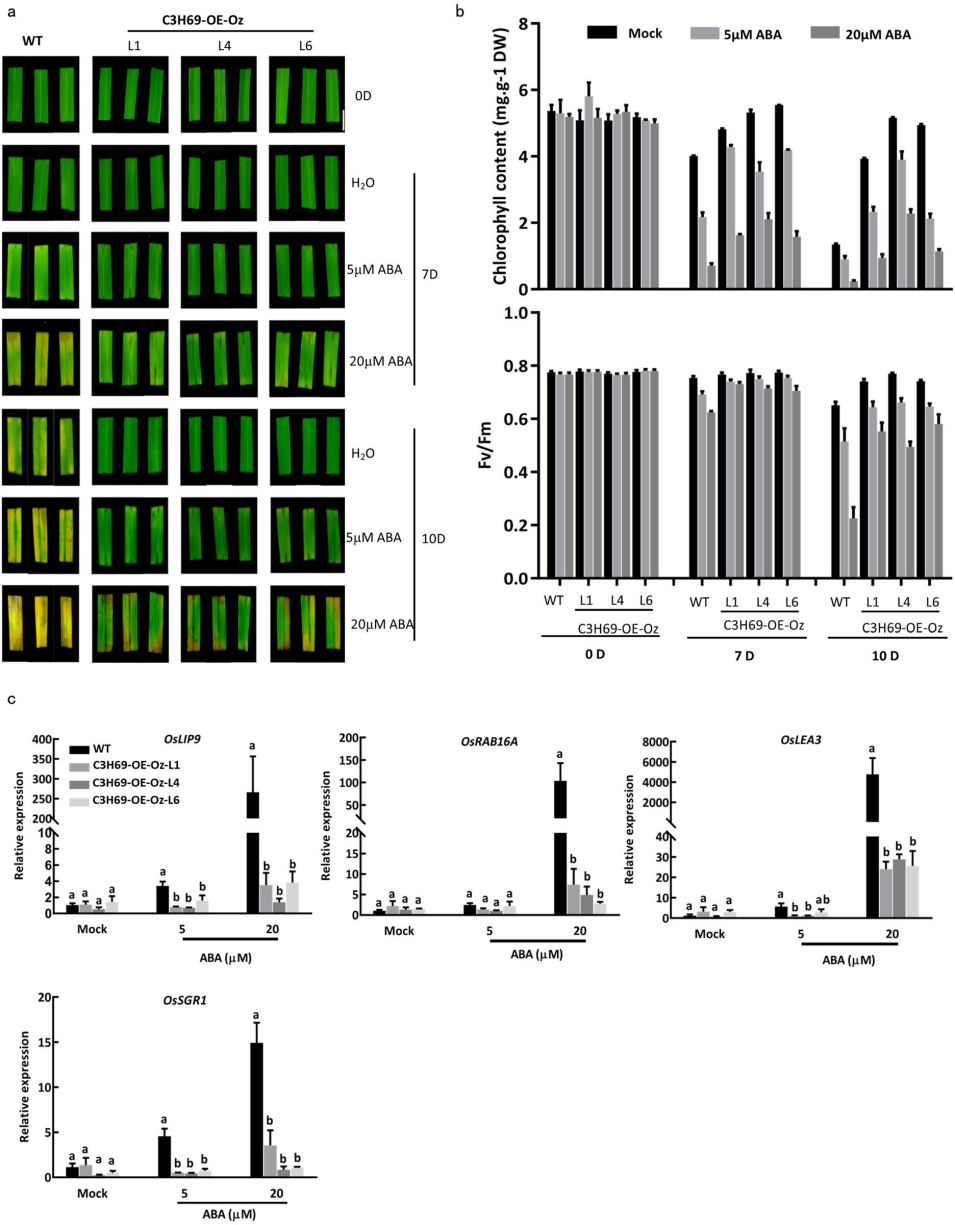
Phenotypic and physiological effects of ABA on dark-induced leaf senescence in transgenic lines (C3H69-OE-Oz) of rice. **a** Phenotype of rice WT and C3H69-OE-Oz lines under exogenous ABA treatment. Excised leaves from rice transgenic lines and WT were treated with 0, 5, 20 μM ABA under darkness. **b** Chlorophyll content and Fv/Fm of rice WT and C3H69-OE-Oz lines under exogenous ABA treatment. **c** Relative expression level of ABA specific response genes in rice WT and C3H69-OE-Oz lines under exogenous ABA treatment. Letters above bars indicate significant difference at *P* < 0.05

Exogenous application of 5 or 20 µM ABA significantly accelerated leaf senescence in rice WT plants, as shown in Fig. 4a, b. In contrast, C3H69-OE-Oz lines of rice were less sensitive to ABA treatment. Leaf Chl content and Fv/Fm of overexpression lines were significantly higher than those of WT plants. Expression levels of three ABA-responsive genes (*OsLIP9*, *OsLEA3*, and *OsRAB16A*) increased 120 to 5000 times in WT plants, but only increased ~4 to 25 times in C3H69-OE-Oz transgenic lines of rice when treated with 20 µM ABA (Fig. 4c). These results demonstrated that *PvC3H69* suppressed ABA-accelerated leaf senescence in rice by downplaying ABA responses.

**Fig. 5:**
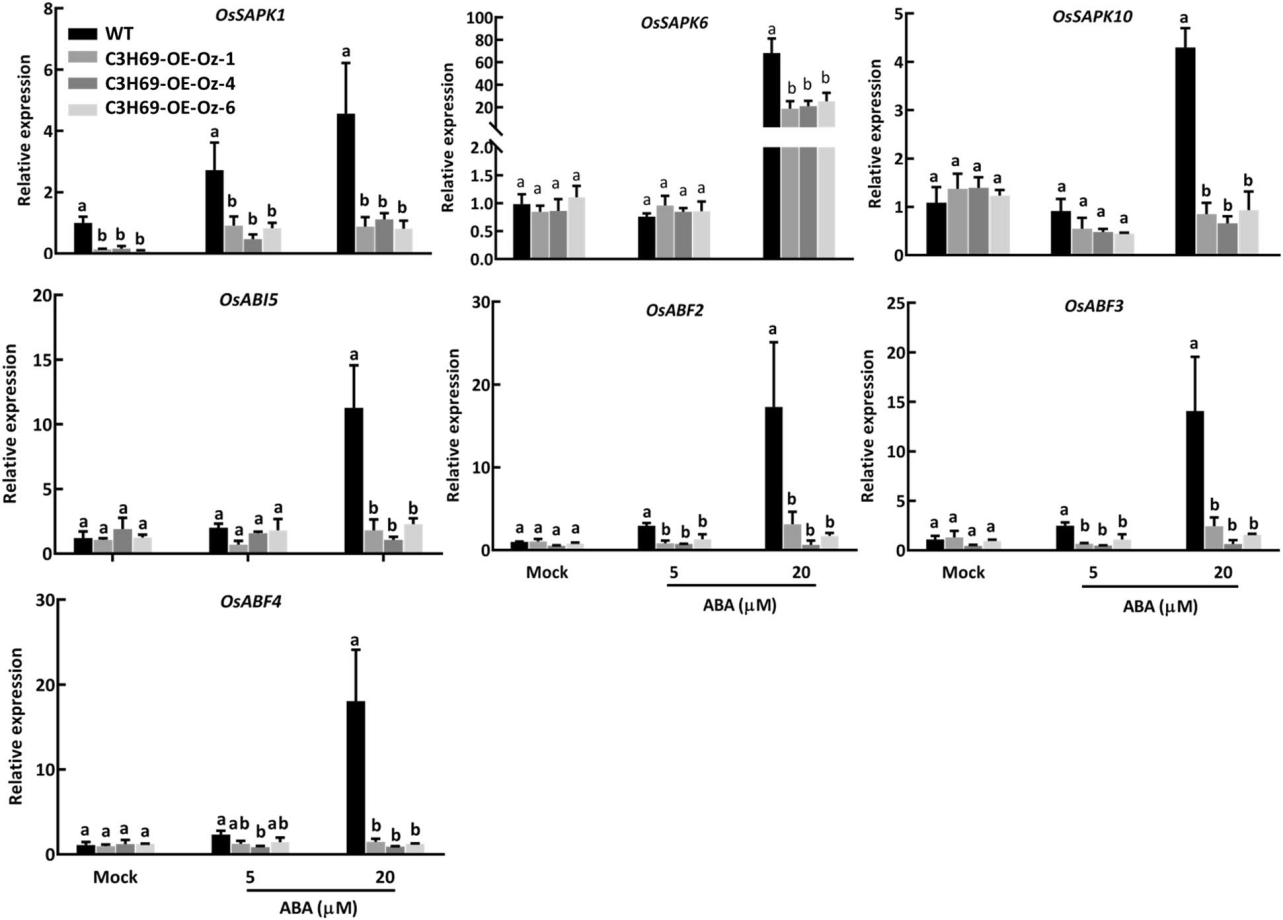
Gene expression patterns in the PP2C-SnRK-ABF signaling in rice transgenic line (C3H69-OE-Oz) and WT plants. Relative expression level of *OsSnRk*, *OsABI5, OsABF2, OsABF3, OsABF4*, and *OsSGR1* in rice C3H69-OE-Oz lines and WT. Letters above bars indicate significant difference at *P* < 0.05

To understand whether *PvC3H69* suppress leaf senescence may involve downregulating ABA signaling, key genes in the PP2C-SnRKs-ABF signaling pathway were further examined. Without ABA treatment (mock), only one *SnRK* family gene, *OsSAPK1*, showed significantly lower expression level in C3H69-OE-Oz transgenic lines compared to WT. With 5 or 20 µM ABA treatment, three (*OsSAPK1, OsSAPK6*, and *OsSAPK10*) out of 10 *SnRK* genes (*OsSAPK1* to *OsSAPK10*) (Fig. S2) and four transcription factor genes (*OsABI5, OsABF2, OsABF3*, and *OsABF4*) showed significantly lower expression levels in C3H69-OE-Oz transgenic lines compared to WT (Fig. 5).

**Fig. 6:**
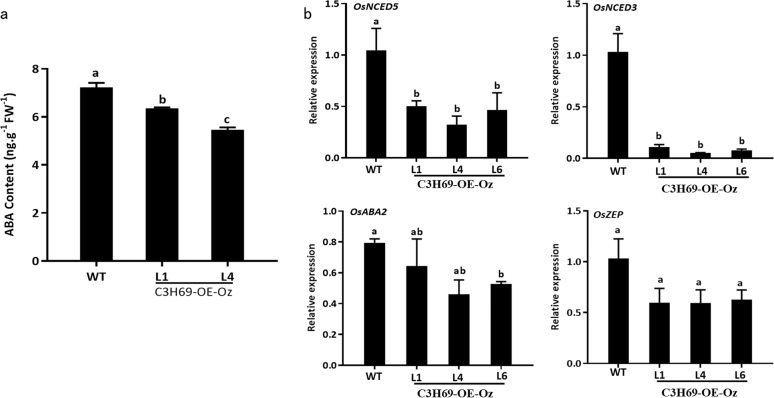
Endogenous ABA content and transcriptional levels of ABA synthesis genes in rice C3H69-OE-Oz lines and WT plants. **a** The net ABA content in rice C3H69-OE-Oz lines and WT. **b** Transcriptional level of ABA synthesis genes (Os*NCED3*, Os*NCED5*, Os*ZEP*, and Os*ABA2*) in rice C3H69-OE-Oz lines and WT. Letters above bars indicate significant difference at *P* < 0.05

We further measured expression levels of four ABA biosynthesis genes (Os*ABA2, OsZEP, OsNCED3, OsNCED5*) and ABA content in leaves of WT and C3H69-OE-Oz lines. As shown in Fig. 6, significantly lower expression levels of *OsNCED3* and *OsNCED5* were detected in C3H69-OE-Oz lines than in WT, and ABA content in C3H69-OE-Oz lines was also significantly lower than that in WT (Fig. 6a, b), indicating that *PvC3H69* suppressed ABA biosynthesis.

### Global gene expression analysis of ABA- and senescence-related genes in *PvC3H69*- overexpression transgenic plants

Comparative analysis of transcriptome of C3H69-OE-Oz and WT leaves exposed to dark for 10 d to induce leaf senescence and those leaves collected prior to dark exposure (0 d) were performed by using Illumina-HiSeq™ 4000. Principle component analysis (PCA) of transcriptomic data showed that differentially expressed genes (DEGs) in WT leaves at 0 d (WT-0), C3H69-OE-Oz leaves at 0 d (C3H69-OE-Oz -0), WT leaves at 10 d of dark treatment (WT-10), and C3H69-OE leaves at 10 d of dark treatment (C3H69-OE-Oz -10) were clustered in separate groups, with DEGs in C3H69-OE-Oz -0 and WT-0 closely related and DEGs in WT-10 and C3H69-OE-Oz -10 were well separated (Fig. S3a). Compared with WT-0, 754 DEGs were upregulated and 580 downregulated in C3H69-OE-Oz -0. A total of 3522 upregulated and 2586 downregulated DEGs in C3H69-OE-Oz -10 compared to WT-10 (Fig. S3b).

Gene Set Enrichment Analysis (GSEA) of Gene Ontology (GO) and Kyoto Encyclopedia of Genes and Genomes (KEGG) were carried out to understand biological functions of DEGs related to dark-induced leaf senescence due to overexpressing *PvC3H69*. Comparative analysis of DEGs between WT-10 and C3H69-OE-Oz -10 found *PvC3H69-*regulated genes and their functions related to the suppression of dark-induced leaf senescence. The most enriched GO term pathways of *PvC3H69-*regulated genes were “photosynthesis”, “thylakoid”, “signaling”, “macromolecular complex”, “response to hormone”, “structural molecule activity”, and “signal transducer activity” (Fig. S4a and Table S2). In particular, the most enriched KEGG pathways were related to hormones and photosynthetic metabolism including “photosynthesis”, “carbon fixation in photosynthetic organisms”, “alpha-Linolenic acid metabolism”, “diterpenoid biosynthesis”, “flavonoid biosynthesis”, “tryptophan metabolism”, “carotenoid biosynthesis”, “Linoleic acid metabolism”, and “plant hormone signaling pathway” (Fig. S4b and Table S3). The results indicated that *PvC3H69* could mainly regulate photosystems and hormone metabolism and signaling to delay dark-induced senescence.

**Fig. 7:**
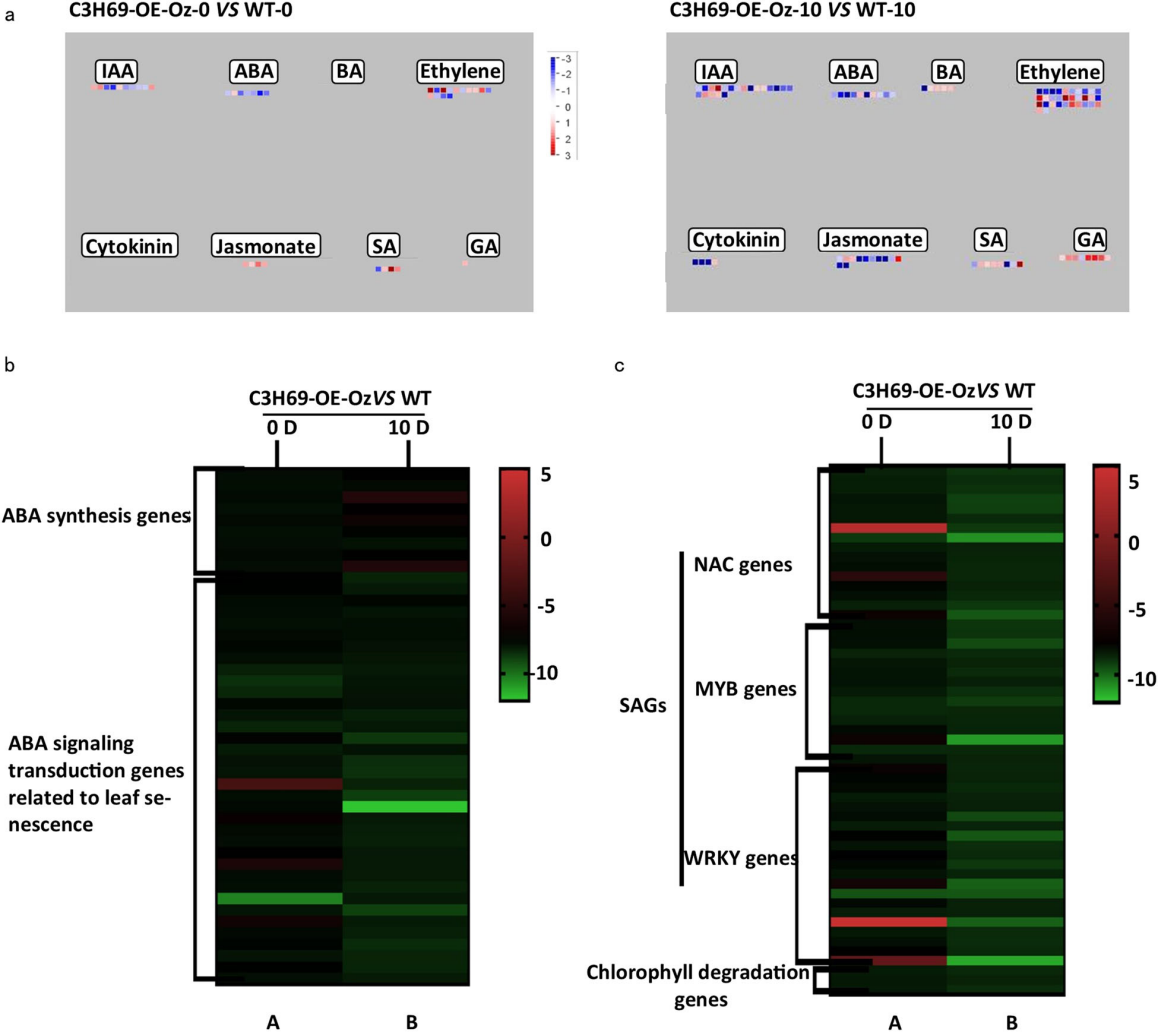
MapMan analysis of “hormone regulation overview” and heatmap of downregulated genes in rice C3H69-OE-Oz lines relative to rice WT plants exposed to 0 and 10 d of dark treatment in ABA pathway and senescence-associated genes through transcriptome analysis. **a** MapMan analysis of “ hormones regulation overview”. **b** Fold change heatmap of ABA synthesis genes and ABA-signaling genes related to leaf senescence in C3H69-OE-Oz -0 (C3H69-OE-Oz transgenic lines prior to dark treatment) vs WT-0 (WT prior to dark treatment) and C3H69-OE-Oz -10 (C3H69-OE-Oz transgenic lines under 10-day dark treatment) vs WT-10 (WT under 10-day dark treatment) transcriptome analysis. **c** Fold change heatmap of senescence-associated genes (SAGs) in C3H69-OE-Oz -0 vs WT-0 and C3H69-OE-Oz -10 vs WT-10 transcriptome analysis

Further analysis of genes in “hormones regulation overview” found DEGs related to ABA, indoleacetic acid (IAA), jasmonic acid (JA), ethylene, salicylic acid (SA), and gibberellic acid (GA). Most DEGs in ABA pathway were downregulated under darkness in plants overexpressing *PvC3H69* (Fig. 7a).

**Fig. 8:**
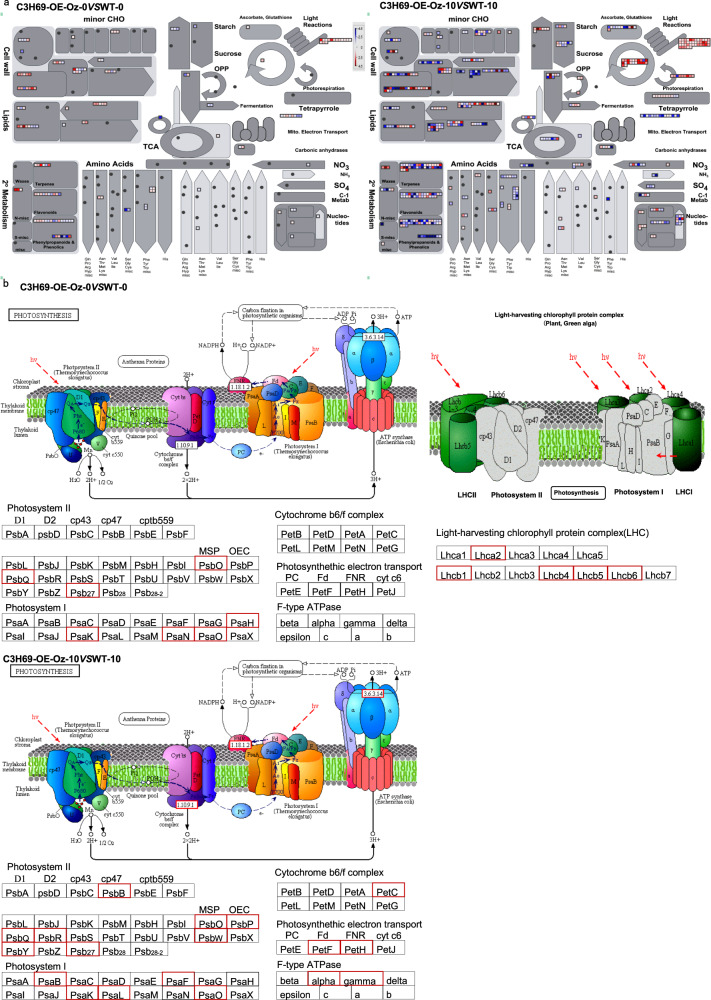
Photosynthetic proteins upregulated in rice C3H69-OE-Oz lines relative to WT plants exposed to 0 and 10 d of dark treatment in transcriptome analysis. **a** MapMan overview DEGs in C3H69-OE-Oz (overexpression plants of *PvC3H69*) vs WT under 0 and 10 d of dark treatment. **b** Upregulated photosynthetic proteins in C3H69-OE-Oz -0 vs WT-0 and C3H69-OE-Oz -10 vs WT-10 transcriptome analysis

Further analysis of DEGs involved in ABA biosynthesis and signaling pathways found nine genes in ABA biosynthesis pathway were downregulated in C3H69-OE-Oz when compared to those in WT by comparing between ‘C3H69-OE-Oz -0 and WT-0’ and between ‘C3H69-OE-Oz -10 and WT-10’. As for DEGs in ABA signaling-related genes, 22 out of 34 were downregulated in C3H69-OE-Oz -0 and all 34 genes were downregulated in C3H69-OE-Oz -10 when compared to WT-0 and WT-10, respectively (Fig. 7b). In addition to ABA-related genes, DEGs encoding for NAC, MYB, and WRKY family transcriptional factor genes known as regulators in leaf senescence were found mostly downregulated in C3H69-OE-Oz plants at 0 d of dark treatment and all of them were downregulated in C3H69-OE-Oz plants at 10 d of dark treatment in comparison to their respective WT plants (Fig. 7c). As for DEGs involved in photosynthesis system, five light-harvesting complexity proteins (Lhca2, Lhcb1, Lhcb4, Lhcb5, and Lhcb6) and seven photosynthesis proteins, including 4 PSI proteins (PsaH, PsaK, PsaN, PsaO) and three PSII proteins (PsbO, PsbQ, and Psb27), were upregulated in C3H69-OE-Oz -0 compared to those in WT-0. DEGs encoding for PSI (PsaB, PsaF, PsaK, PsaL, and PsaO) and PSII components (PsbB, PsbO, PsbP, PsbQ, PsbR, PsbW, PsbY, and Psb27) were upregulated in C3H69-OE-Oz -10 compared to those in WT-10 (Fig. 8a, b).

The expression of selected DEGs was confirmed with qRT–PCR analysis, including four ABA signaling-related genes (*SnRK2*, *ABI5*, *NAC103*, and *NAC58*), three other hormonal signaling pathways (*ARR*, *ERF103*, and *ORE1*), and two chlorophyll degradation genes (*SGR* and *NOL*). The relative expression of these nine genes in qRT–PCR analysis corresponded to those generated from the RNA-Seq, supporting the reliability of RNA-Seq results (Fig. S5).

## Discussion

The CCCH-type zinc finger family includes multiple genes, and *PvC3H69* cloned from switchgrass in our study was found to be a homology of *ZmC3H38* and *OsTZF1*^24^, but exhibited unique characteristics from the homologs in maize and rice. PvC3H69 was located in nucleus, but had no transcriptional activity (Fig. 1a–d), suggesting it is not a transcriptional factor. Other CCCH proteins in rice and Arabidopsis with functions in DNA binding, RNA binding, and mRNA turnover or silencing can shuttle between the nucleus and cytoplasm foci under different stresses^9,26^. TZF1 proteins have been found to directly interact with stress regulators, such as RD21A and PR5 or the mRNA of Ank-β^9,16^. However, whether PvC3H69 has mRNA or protein binding function deserves further investigation.

Although CCCH genes are known to regulate leaf senescence, different genes in the CCCH family were found to have distinct functions with *AtKHZ1* and *AtKHZ2* being characterized as a positive regulator inducing leaf senescence in Arabidopsis^6^ and *OsDOS* and *OsTZF1* as a negative regulator of leaf senescence in rice^7–9^. In our study, *PvC3H69* acted as a negative regulator, which suppressed both natural senescence associated with leaf aging and dark-induced leaf senescence, as manifested by the maintenance of greater chlorophyll content, photochemical efficiency, and net photosynthetic rate in plants overexpressing *PvC3H69* (Figs. 2 and 3). Furthermore, transcriptomic analysis of plants overexpressing *PvC3H69* found that a large number of genes involved in the light-dependent process of photosynthesis, including light-harvesting complex proteins (Lhca2, Lhcb1, Lhcb4, Lhcb5, Lhcb), PSI proteins (PsaH, PsaK, PsaN, PsaO) and PSII proteins (PsbO, PsbQ, Psb27) were upregulated in natural senescent leaves and those in PSI systems (PsaB, PsaF, PsaK, PsaL, PsaO) and PSII components (PsbB, PsbO, PsbP, PsbQ, PsbR, PsbW, PsbY, Psb27) were upregulated in dark-induced senescent leaves (Fig. 8). In contrast, DEGs encoding NAC (NAC58, NAC103 et al.), MYB-like, and WRKY (WRKY45 et al.) family transcriptional factor genes which are known regulators in leaf senescence were downregulated in C3H69-OE plants in comparison to WT plants (Fig. 7c). The combined physiological and transcriptomic data provided strong evidence that *PvC3H69* acted as a repressor for leaf senescence, which helped to sustain or maintain photosynthesis by enhancing light-harvesting and photochemical capacity in both PS I and PS II of photosynthesis.

The underlying molecular mechanisms and key regulatory pathways for CCCH suppressing leaf senescence are yet to be fully understood. The delay in stress-induced leaf senescence by *OsTZF1* has been associated with regulating stress-related genes (i.e., *AK112082*, *JAZ1*, *Ferritin*, *MT-type1*, *ChaC-like*)^8,9^. In our study, DEGs by *PvC3H69* were highly enriched in hormone metabolism and signaling pathways, with most genes in ABA biosynthesis and signaling being downregulated significantly by overexpressing *PvC3H69* (Fig. 7b). Moreover, transgenic rice plants overexpressing *PvC3H69* exhibited lower sensitivity of leaf senescence to ABA treatment compared with WT plants (Fig. 4a, b). These results indicated that function of *PvC3H69* is involved in ABA.

ABA-induced leaf senescence involves activation of ABA biosynthesis genes, such as *NCEDs*, *ABA2*, *AAO3*, and ABA signaling genes, such as *ABI5*, *ABF2*, and *ABF3*^18–22^. *OsNECD3* and *OsNECD5* are involved in xanthophyll cleavage for ABA biosynthesis^27,28^. The relatively lower content of ABA in C3H69-OE plants could be resulted from the downregulated transcript level of *OsNECD3* and *OsNECD5*. Consistently, global genes expression analysis showed that nine genes in ABA biosynthesis, including *OsNCED3* and *OsAAO3* were downregulated, indicating that PvC3H69 could be a negative regulator for ABA biosynthesis (Fig. 6a, b). In ABA signaling, PP2C-SnRK-ABF regulatory model is considered as the core pathway that is required for ABA-triggered Chl degradation^21,22^. In this model, PYL as a receptor can accept ABA signal and form PYL-ABA bound complexity, which leads to PP2C inactivated and released the repressed SnRKs (mainly SnRK2s); the activated SnRKs can phosphorylate ABA-response binding factors (ABFs)^29,30^. *SnRK2* genes are plant-specific serine/threonine kinases involving in plant responses to abiotic stresses and ABA-dependent plant development^31,32^. In rice, there are 10 SnRK proteins in SnRk2.0 family and are designated as SAPKs (stress-activated protein kinase). Among them, OsSAPK8, OsSAPK9, and OsSAPK10, were also activated by ABA^33^. OsSAPK1 can be upregulated by osmotic stressors and it has been reported that OsSAPK1 can be directly regulated by OsNAC2 through an ABA-dependent pathway^34^. Ectopic expression of *SAPK6* (*OSRK1*) in tobacco confers reducing ABA sensitivity^35,36^. In our study, three *OsSAPKs*, *OsSAPK1*, *OsSAPK6*, and *OsSAPK10* were downregulated by overexpressing *PvC3H69*. All these results suggested that *PvC3H69* could interrupt both ABA biosynthesis and signaling, thereby suppressing leaf senescence in C3H69-OE plants.

Further evaluation of the relative transcript changes of ABA-biosynthesis and ABA-signaling genes in leaves in C3H69-OE lines and WT exposed to dark found that *PvC3H69* caused downregulation of all genes in the ABA signaling pathway in a greater magnitude compared to those genes for ABA biosynthesis, suggesting ABA signaling could be more sensitive to negative transcriptional control of *PvC3H69*. In addition, ABI5 and ABF2/3/4 are key phosphorylating substrates for SnRKs which belong to basic leucine zipper (bZIP) TFs and AREB binding TFs, respectively, and positively affect leaf senescence by directly anchoring the promoter of *SAGs* including *SGR*, *NYC1*, and *ABR*^5,22^. The reduced transcript level of *SnRKs*, *ABI5*, and *ABF2/3/4* in C3H69-OE plants treated with ABA (Fig. 5) indicated that *PvC3H69* could negatively regulate ABA-induced leaf senescence mainly through PP2C-SnRK-ABF signaling pathway.

**Fig. 9:**
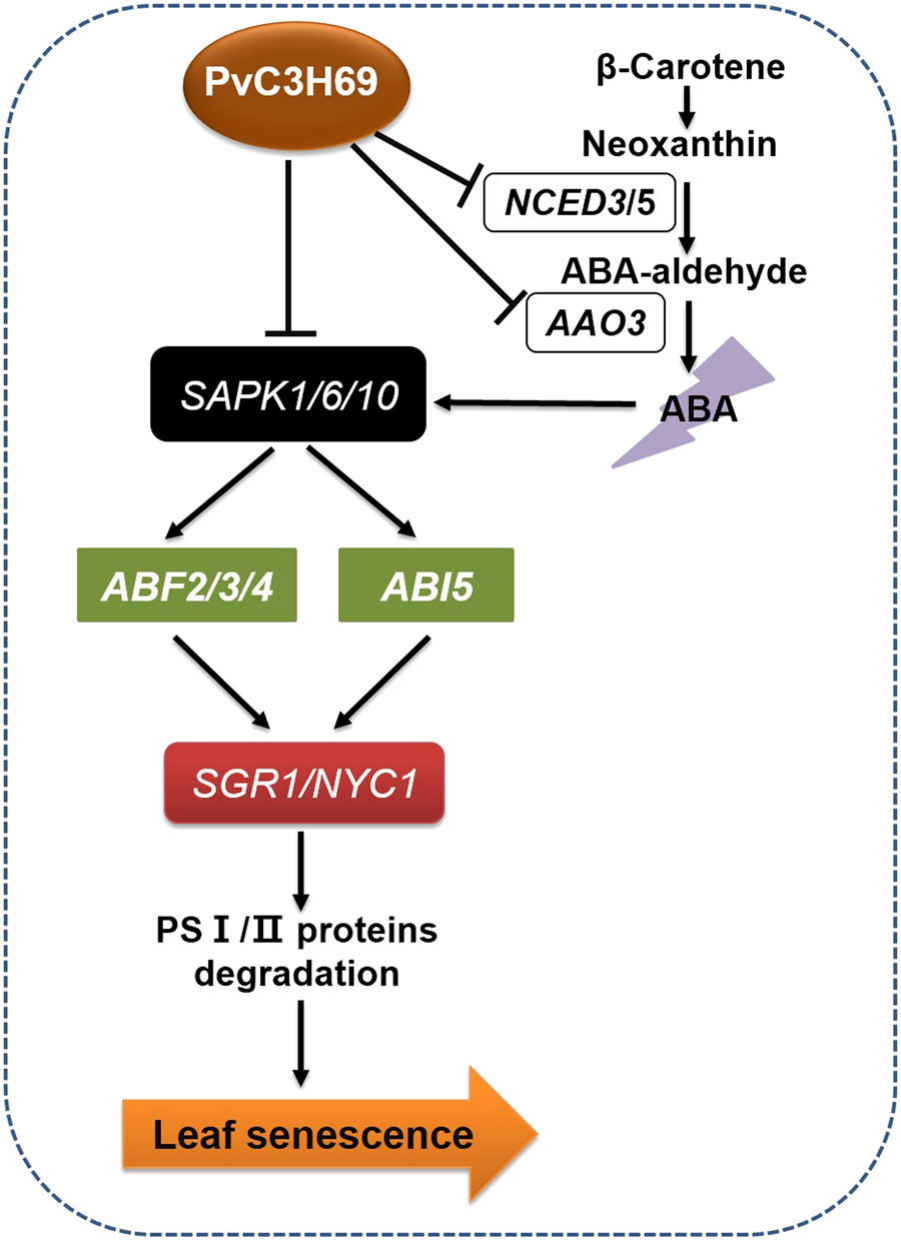
Proposed pathways of *PvC3H69* regulating ABA-mediated leaf senescence: *PvC3H69* could repress the expression of *NCED3/5* and *AAO3*, and then repressed ABA synthesis. In ABA transduction signaling, *PvC3h69* could also repress the expression of *SnRK1/6/10* and further decrease the phosphorylationof *SnRK1/6/10* on *ABF2/3/4* and *ABI5*; and then further surpressed the expression of *SGR1/NYC1* and PSI/PSII protein degradation, then delay the leaf senescence

In summary, overexpression of *PvC3H69* in rice or its native plants resulted in a stay-green phenotype, strongly suggesting that *PvC3H69* was a negative regulator in leaf senescence. *PvC3H69* could facilitate the stay-green phenotype or delayed leaf senescence mainly by upregulating light-dependent process of photosynthesis, including light-harvesting complex proteins, PSI proteins, and PSII proteins and repressing ABA biosynthesis and signaling genes and senescence-associated genes such as *NCED3/5*, *AAO3*, *SnRK1/6/10*, *ABF2/3/4*, *ABI5*, *SGR*, and *NYC1* (Fig. 9). *PvC3H69* suppression of ABA-mediated leaf senescence with leaf aging or induced by darkness was mainly through regulating PP2C-SnRK-ABF signaling pathway. Future research could identify its upstream regulatory factors and further confirm the functions of *PvC3H69* in leaf senescence induced by other abiotic stress, such as heat, drought, and salinity in order to improve plant tolerance to diverse environmental stresses.

## Experimental procedures

### Gene cloning and vector construction

The full-length gene of *PvC3H69* (Phytozome accession no.: Pavir.J04795.1), with 394 amino acids without intron was amplified from the gDNA of a lowland ecotype ‘Alamo’ switchgrass^37^. The gene was firstly cloned into the Gateway entry vector pENTR/D (Invitrogen). It was subcloned into p2GWF7.0^38^, pGBKT7 (Invitrogen), and pVT1629^37^ through LR reaction (Invitrogen). The primers used for *PvC3H69* cloning are listed in Table S1.

### Observation of subcellular localization of PvC3H69-GFP

The *PvC3H69* was subcloned into a modified gateway-compatible P2GWF7.0 vector to put *PvC3H69* in fusion with *GFP*. By polyethylene glycol (PEG)-mediated Arabidopsis protoplast transformation^39^, the *PvC3H69-GFP* fusion gene was overexpressed in Arabidopsis protoplasts. DAPI was used to stain the nucleus, and the GFP signals were detected under a Zeiss LSM 780 laser scanning confocal microscope (Carl Zeiss SAS, Jena, Germany).

### Transactivation assay

*PvC3H69* was subcloned into the BD vector pGBKT7 to fuse *PvC3H69* with the DNA-binding domain of GAL4. The pGBKT7-*PvC3H69* and the control vector pGBKT7-*GUS* (*UiDA gene*) were then transformed into the yeast strain Y2HGold (Clonetech), separately. The pGBKT7-*PvC3H72* was used as a positive control^25^. The transformed positive clones grown well on SD/-Trp were then grown on plates containing SD/-Trp-Ade-His and SD/-Trp-Ade-His + 25 mM 3-AT for auto-transactivation assay.

For the transcriptional activity assay of *PvC3H69* in plant cells, *PvC3H69* was cloned into the 35S promoter-droven pZB370 vector to fuse with the yeast GAL4 DNA-binding domain (GAL4BD) as effector (pZB369-*PvC3H69*), while the vector without the target gene was used as the negative control. As a positive control, PvC3H72 has been reported to be a transcriptional activator^25^. The internal control vector was pZB371-Luciferase under driven of *35S* promoter as well. The reporter vector (pZB370-GUS) was constituted of four copies of GAL4 DNA-binding sites (GAL4(4x)-D1-3(4x)) to drive the *GUS* (*UidA*) reporter gene. Three plasmids (effector, reporter, and internal control) were co-transferred into Arabidopsis protoplasts at the ratio of 5:4:1. The transcriptional ability was assessed by the GUS/LUC ratio. Three biological replicates were included for each combination.

### Plant transformation and verification

Switchgrass genetic transformation followed the protocol described in Xu et al.^37^. Embryogenic calluses of a selected line ‘HR8’ from switchgrass lowland ecotype ‘Alamo’ were infected with Agrobacterium tumefaciens strain ‘AGL1’ harboring the binary vector pVT1629-*PvC3H69* with the target gene under driven of the maize ubiquitin promoter, and selected the putative transgenic lines on 50 mg L^−1^ hygromycin (Sigma). GUS staining and regular PCR for the presence of the T-DNA fragment of transgenic lines were the same as reported previously^25^.

Nipponbare (*Oryza sativa japonica cvNipponbare*) was used in this study. The transformation system was referred to Toki et al.^40^. Leaves of transgenic lines were stained with GUS solution. DNA was extracted from the C3H69-OE plants for PCR detection. RNA was extracted from the leaves of transgenic lines as a template for determining the transcriptional level of *PvC3H69*.

### Plant growth conditions and dark treatment

Switchgrass transgenic lines and WT were grown in the green house with temperatures set at 28 °C/22 °C, day/night with a 14-h light/10-h darkness. The plants were watered twice a week. In order to induce leaf senescence, the middle 1/3 part of whole detached full-expand leaves from 3-month-old seedlings were cut into 3-cm segments and placed in a dark room with air temperature controlled at 28 °C. Leaf samples were collected at the time points of 0, 10, and 15 day for phenotypic and physiological analysis. Five-month-old plants were used for phenotypic and physiological analysis of natural senescence.

T3 seeds of WT and transgenic rice ‘Nipponbare’ were sterilized and germinated on 1/2 Murashige and Skoog (MS) medium, which were transferred to bucket filled with IRRI (International Rice Research Institute) nutrient solution in a growth chamber controlled at 30 °C during the day and 25 °C at night with 16-h light/8-h of darkness. The middle 1/3 part of whole detached full-expand leaves of 6-week-old plants were cut into about 3-cm fragments for dark treatment with air temperature controlled at 25 °C. Leaf fragments were collected at the time points of 0 d (day), 10 d, and 13 d for further phenotype and physiological index measurement. For ABA treatment, leaf fragments (same cut with the dark treatment) of WT and transgenic rice were soaked in different concentration ABA solutions (0, 5, and 20 µM) under darkness. Leaf samples were collected at the time points of 0, 7, and 10 d for phenotypic, physiological analysis. Samples at 7 day were used for qRT–PCR analysis.

### Physiological analysis of transgenic switchgrass and rice

Leaf net photosynthetic rate (Pn) was measured with a LI-6400 system (LI-COR Inc., Lincoln, NE) equipped with a standard 2 × 3 cm^2^ leaf chamber with light-emitting diodes as a light source. The measurements were taken at the PAR of 800 µmol m^−2^ s^−1^ and flow rate of 500 µmol s^−1^. The block temperature was set to 25 °C for optimal temperature. For leaf photochemical efficiency (Fv/Fm), leaves were put in a 30 min dark-adaptation period and measured using a fluorescence induction monitor (OPTI-Sciences, Hudson, USA) as the ratio of variable (Fv) to maximum (Fm) fluorescence. Chlorophyll content (Chl) was measured by extracting chlorophyll from 0.1 g fresh leaves in 10 ml dimethyl sulfoxide (DMSO) under dark for 4 d and measuring the absorbance at 663 and 645 nm. The blades were dried in an oven at 80 °C for dry weight. Chl content was calculated using the formula described in Arnon^41^.

For endogenous ABA content analysis, leaves of 4-week-old rice seedlings were used to measure the endogenous ABA content. ABA was extracted from 500 mg frozen leaf powder according to the method reported by Krishnan et al.^42^. The samples were suspended with extraction buffer (methanol:water:acetic acid, 80:19:1, v/v/v) and shake for 12 h at 4 °C, then centrifuged at 14,000 rpm for 20 min. The supernatant was collected in a new tube, pellet were reextracted with 500 μl extraction buffer, shaken for 4 h at 4 °C under darkness, then centrifuged at 14,000 rpm for 20 min at 4 °C. Two tubes of supernatant were mixed and dried using centrifugal vacuum concentrator and then dissolved in 300 µL methanol. The resulted supernatant ABA concentration was determined by high-performance liquid chromatograph using SCIEX-6500Qtrap mass spectrometer (HPLC-MS/MS; Aglient1290, Agilent, USA).

### Rice transcriptome analysis and qRT–PCR analysis

For transcriptome analysis, total RNA was extracted from the leaves of 4-week-old rice WT and overexpressing *PvC3H69* (C3H69-OE) plants exposed to dark treatment for 10 d and prior to dark treatment (0 d) according to the manufacturer’s instructions using RNA extract kit (Invitrogen, Carlsbad, CA, USA). mRNA was enriched by Oligo (dT) beads. Then the enriched mRNA was fragmented into short fragments and reverse into transcripted into cDNA. cDNA were purified with QiaQuick PCR extraction kit (Qiagen, Venlo, The Netherlands). Three independent biological replicates were conducted for the WT and C3H69-OE plants. The differently expressed genes in WT and C3H69-OE plants were classified functionally using the biological process category of Rice Gene Ontology (ftp://ftp.ensemblgenomes.org/pub/plants/release-39). Significant interactors were determined using a two-sample analysis (*t* test).

For qRT–PCR analysis, total RNA extraction, first-strand cDNA synthesis, PCR reaction, and data analysis were the same as our previously reported by Xie et al.^25^. Primers for qRT–PCR are listed in Table S1.

### Statistical analysis

Leaf senescence (including dark-induced and age-induced) and ABA treatment effects and variations among WT and transgenic plants for physiological parameters and gene expression levels were analyzed using SAS v9.2 (SAS Institute, Cary, NC, USA). Mean data were separated Fisher’s protected LSD at the probability of 0.05.

## Supplementary information

Supplementary Figures S1-S5

Supplemental Table 3

Supplemental Table 1

Supplemental Table 2
