# Supplementary material for: CCCH protein-PvCCCH69 acted as a repressor for leaf senescence through suppressing ABA-signaling pathway
Source: Hortic Res. 2021 Jul 7;8:165. doi: 10.1038/s41438-021-00604-0 (PMC8263708; doi:10.1038/s41438-021-00604-0)
Supplement: Supplementary file 1 — Supplementary Figures S1-S5 [file 41438_2021_604_MOESM1_ESM.docx]

**Supporting Information**


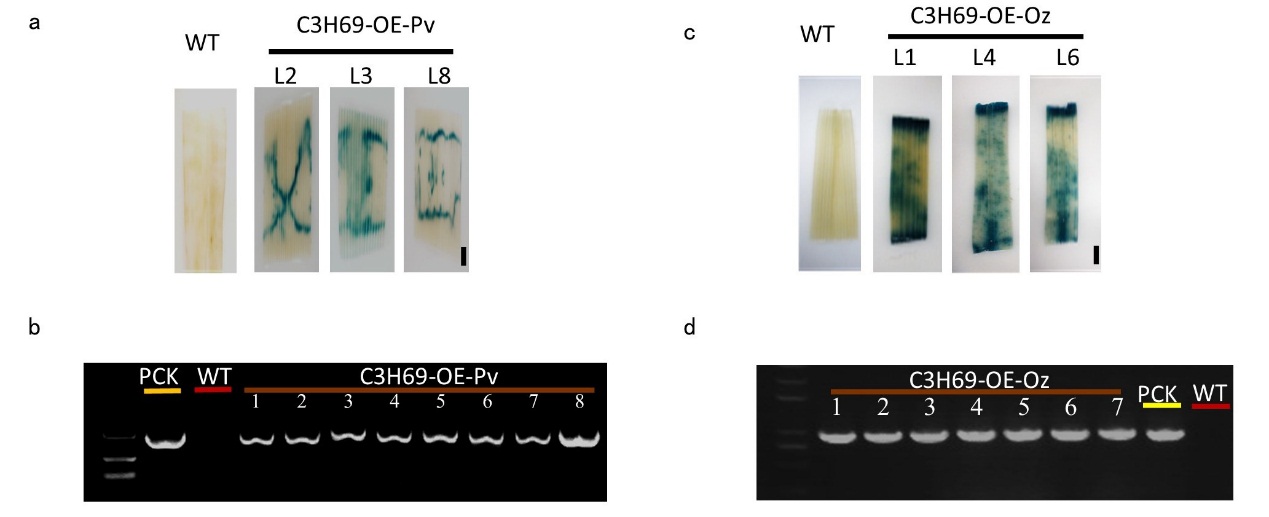


**Figure S1: GUS staining and PCR confirmed of** C3H69-OE **plants in switchgrass and rice.**a, GUS staining of switchgrass WT and C3H69-OE-Pv plants (L2, L3, L8). b, *Hygromycin* gene amplification in switchgrass WT and C3H69-OE-Pv plants using PCR. c, GUS staining of rice WT and C3H69-OE-Oz plants (L1, L4, L6). d, *Hygromycin* gene amplification in rice WT and C3H69-OE-Oz plants using PCR. Bar=200 μm.


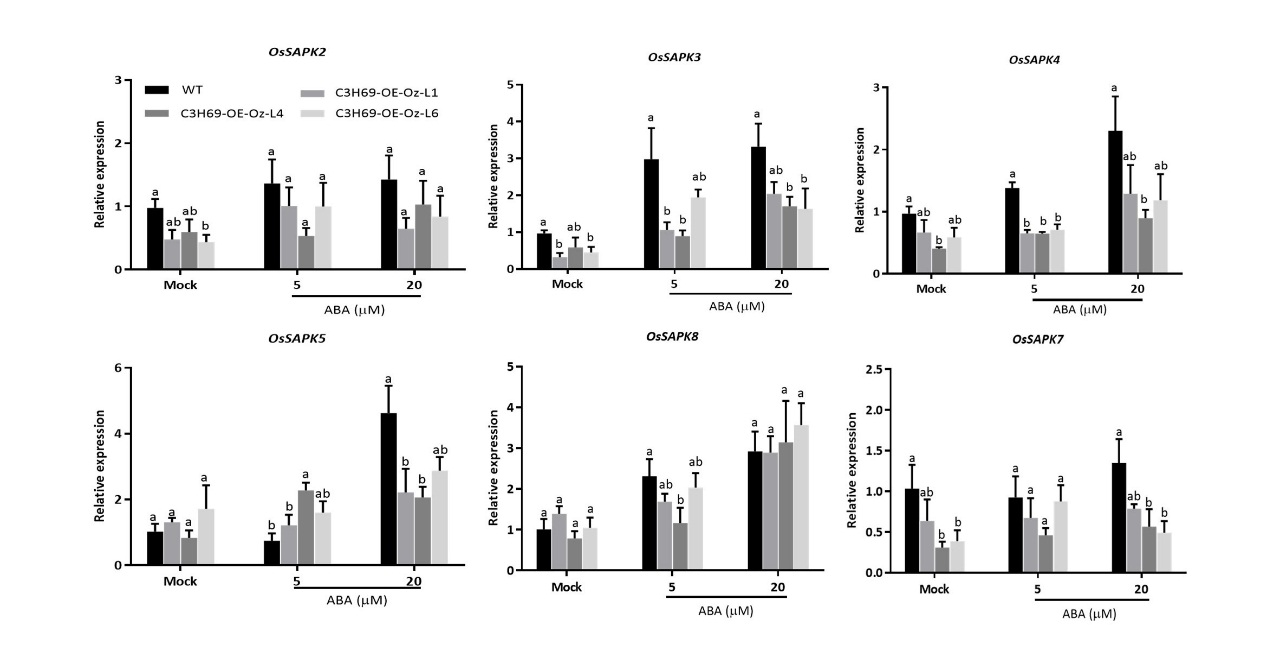


**Figure S2: Transcriptional level of other *OsSnRK* genes in rice under ABA treatment.**


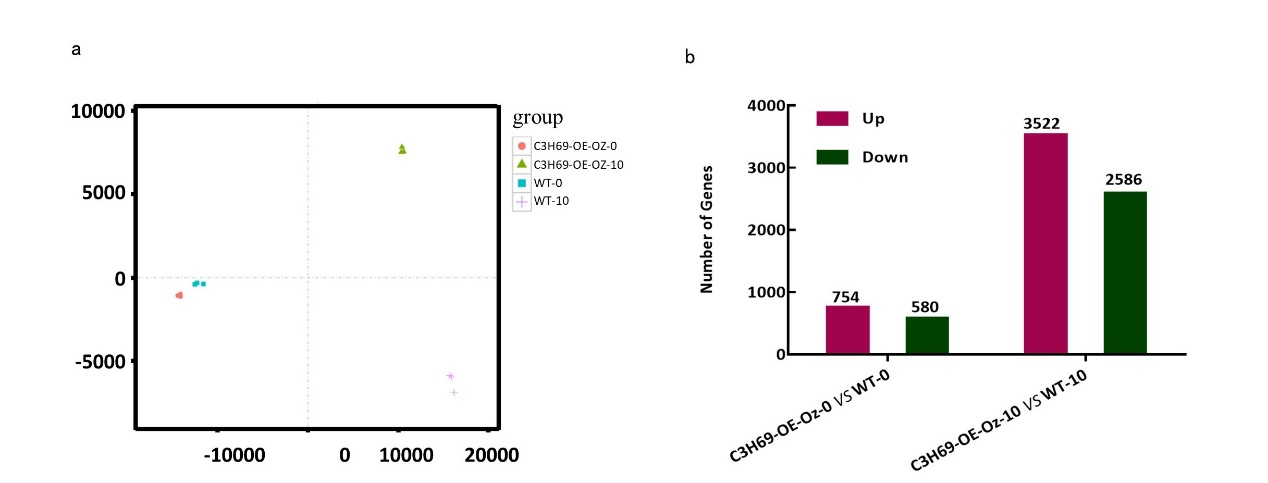


**Figure S3: PCA analysis and number of DEGs through transcriptome analysis in rice.**

a, PCA analysis of tanscriptome analysis. Red point is C3H69-OE-Oz -0 (C3H69-OE-Oz lines before dark treatment),green triangle is C3H69-OE-Oz -10 (C3H69-OE-Oz lines exposed to 10 days dark treatment), blue square is WT-0 (WT before dark treatment), black cross is WT-10 (WT exposed to 10 days dark treatment).b, Number of genes of up-regulated and down-regulated in C3H69-OE-Oz -0 *vs* WT-0 and C3H69-OE-Oz -10 *vs* WT-10. Purple is represent for up-regulated, atrovirens is represent for down-regulated.


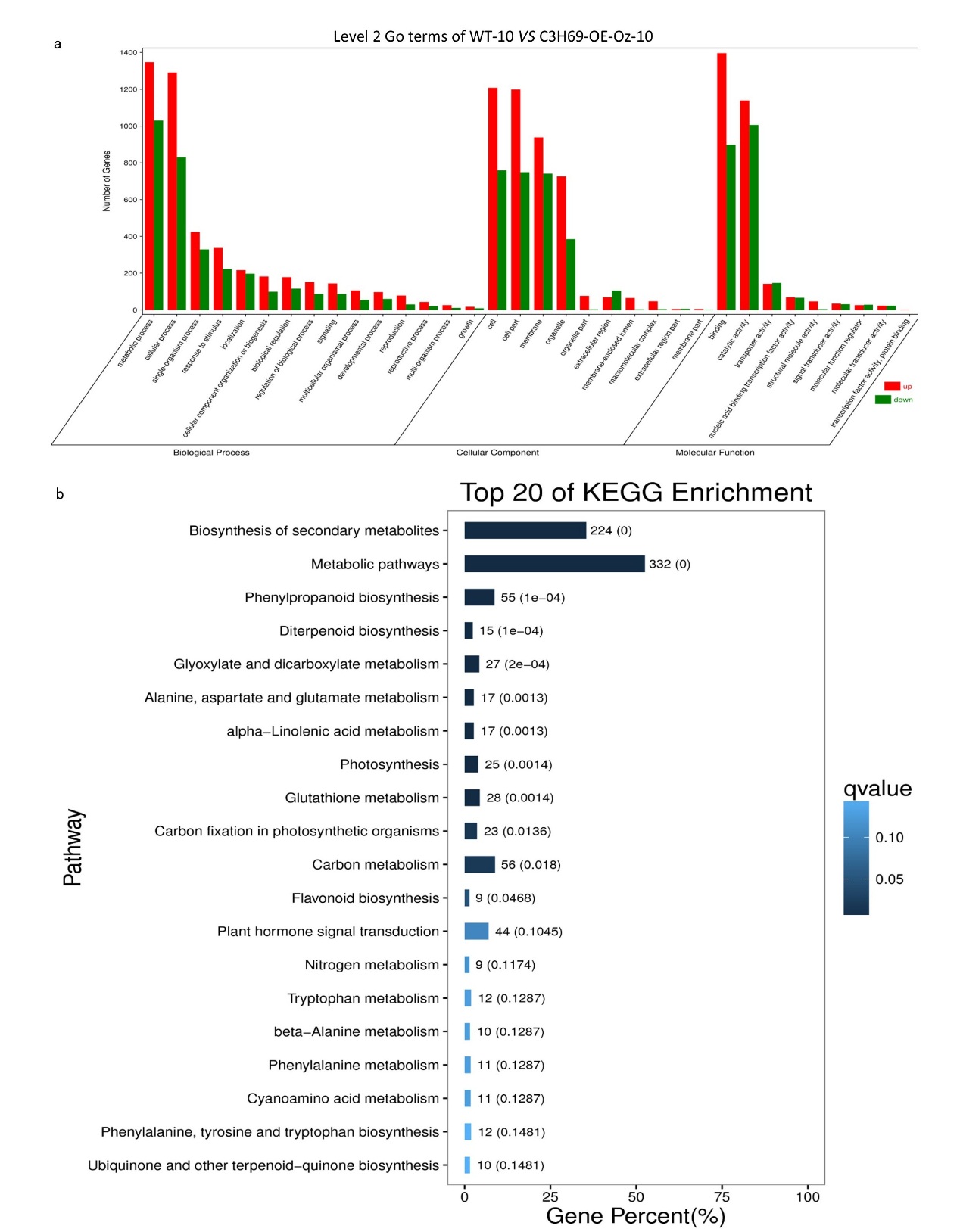


**Figure S4: Differentially-expressed genes (DEGs) in GO and KEGG pathway for rice WT and transgenic line (**C3H69-OE-Oz**) exposed to 10-d of dark treatment.**a, DEGs in GO pathway for WT -10 *vs.* C3H69-OE-Oz -10; b, DEGs in KEGG pathway for WT-10 *vs.* C3H69-OE-Oz -10.


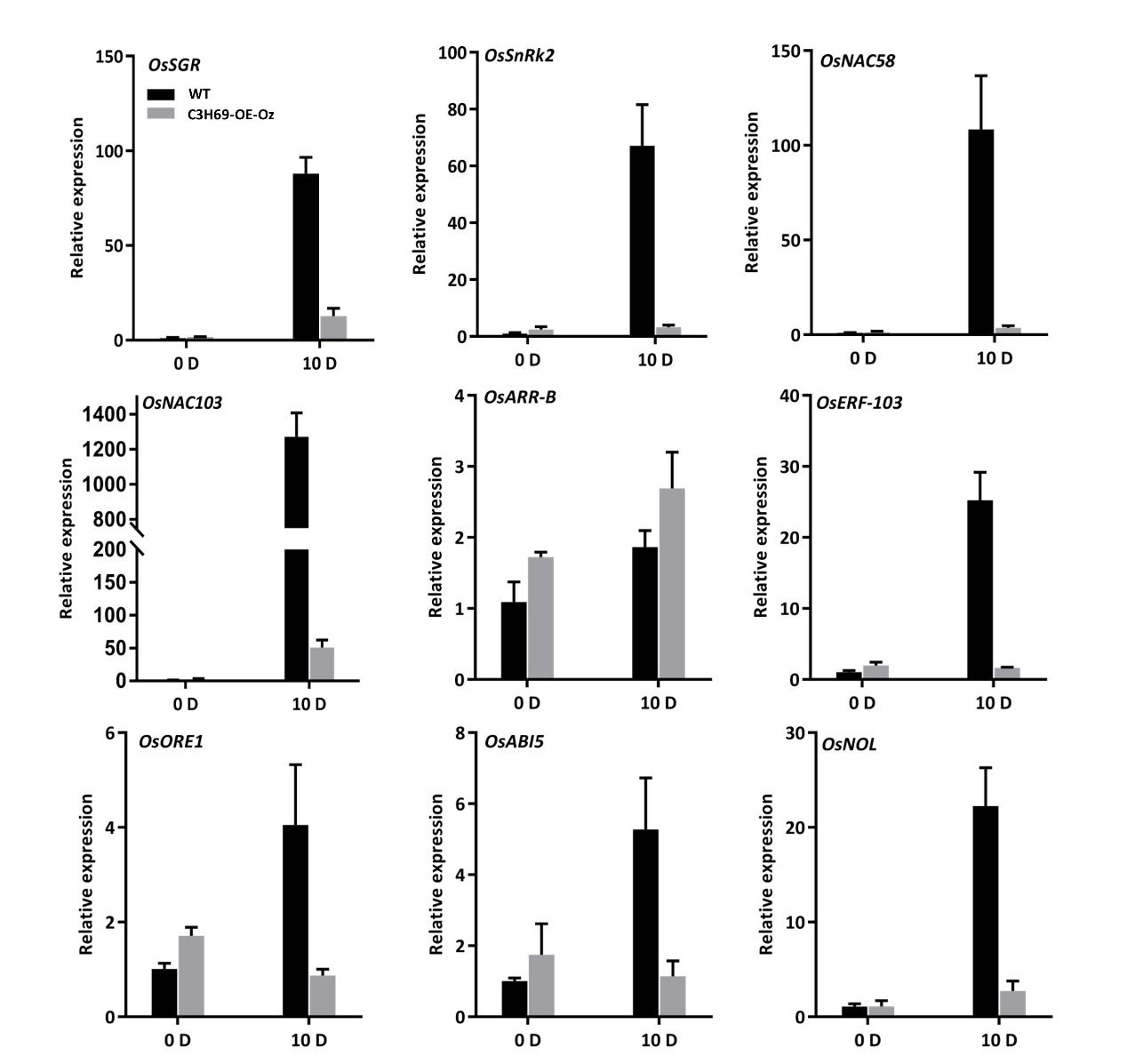


**Figure S5: DEGs were confirmed with qRT –PCR to validate the RNA-seq data.**
