# Supplementary material for: CCCH protein-PvCCCH69 acted as a repressor for leaf senescence through suppressing ABA-signaling pathway
Source: Hortic Res. 2021 Jul 7;8:165. doi: 10.1038/s41438-021-00604-0 (PMC8263708; doi:10.1038/s41438-021-00604-0)
Supplement: Supplementary file 2 — Supplemental Table 3 [file 41438_2021_604_MOESM2_ESM.docx]

**Table 1 Primers used for qRT-PCR**

| **Gene name** | **Forward Primer Sequence 5’-3’** | **Reverse Primer Sequence 5’-3’** |
| --- | --- | --- |
| *OsZEP* | GGATGCCATTGAGTTTGGTT | TGGCTGACTGAAGTCTCTCG |
| *OsNCED3* | CCCCTCCCAAACCATCCAAACCGA | TGTGAGCATATCCTGGCGTCGTGA |
| *OsNCED5* | ACATCCGAGCTCCTCGTCGTGAA | TTGGAAGGTGTTTTGGAATGAACCA |
| *ABA2* | CTGAAAGGTGTGGATCTGCT | ACTAGAGTACCAATTGTGCCT |
| *OsUbiq* | ACCACTTCGACCGCCACTACT | ACGCCTAAGCCTGCTGGTT |
| *OsABI5* | GGAGATGAGCAAGAACGTGA | TAGCTCTTCCAAGGTGAGGG |
| *OsSGR1* | AGAGGGATTAATTAGCCGCG | CATGGTCGAAGTAGCAGCAG |
| *OsNYC1* | AAAACCAAGGAACCCAACCC | CTCCTCCCCGCCGTTATC |
| *OsABF2* | TCACACTCCTCTCTTCTCCG | AACTCCATCTCCTCTCCCAC |
| *OsABF4* | TTCGATTGGTTTGGTTGGGT | GCTCTGGAACTCGTCGAAC |
| *OsABF3* | CCCCACTTCCCTTTTCTTCC | CAGCAGCTCATCCATGTTCA |
| *OsORE1* | AACCCGGAGATCTCGTCATC | CTTGGTAGATGCCTCGATCG |
| *OsNOL* | CTTGGCCAACTACGCATTTG | ACAAGAAGCAAATCACGGGT |
| *OsB-ARR* | ACTTCACGGTGTCTCCCTT | GAACATCATCCTTCCTCGCA |
| *OsNAC58* | TGCTGCTCCACTCTAACATG | GACGACCTAGCTAGTCCACA |
| *OsNAC103* | ACTCAACCACCAGATGCTTC | ATCATCGTCGTCCATGCTTC |
| *OsERF* | CCTGGAGCCGTACATGAAAT | AACCCCTCAGTAGAACTCGG |
| *OsSAPK1* | AGCATGGATCTCGACGACAT | CCCCGCTACTTCAGTCTTCA |
| *OsSAPK2* | TCCAAATTCTCCGGGCAGAT | GAGCAGCACACTCCAAGAAG |
| *OsSAPK3* | CGCCGATGAGTATGACACCT | ACGCATATCATTTGGTCTGGC |
| *OsSAPK4* | TCAGACGAGGAGGAGAAGGA | CGCAGTTCCATAGTAAGCCG |
| *OsSAPK5* | CCATCGTCGTCCATACTCCA | ATCAGTGCAGATGGGGTTGA |
| *OsSAPK6* | AGGACGAGTACACCAAGCAG | CCCCACAACTACCACAAAGT |
| *OsSAPK7* | TGAGTACGACAAGCAGGTGA | TGACCATCAGCTGCCACATT |
| *OsSAPK8* | ACAAGAGAACTGAAGGGGCA | CCACGGCAAGAAACTAAGCA |
| *OsSAPK9* | AAGCAACAATTCCAGCAGCA | TCAAGGTCAAGGTCCGAGTC |
| *OsSAPK10* | AATGATGGCCTTGACCTCGA | ATCTGCCTGTCTGCTTGCTA |
| *OsLEA3* | GCCGTGAATGATTTCCCTTTG | CACACCCGTCAGAAATCCTCC |
| *OsLIP9* | TGGAATTTGGAAGTGTTTGGC | CCCACACGAAACACAAACTTC |
| *OsRAB16A* | CATGGACAAGATCAAGGAGAAGC | CTTATTATTCAGGAAGGTGACGTGG |

**Table 2 Primers used for PCR**

| **Gene name** | **Forward Primer Sequence 5’-3’** | **Reverse Primer Sequence 5’-3’** |
| --- | --- | --- |
| ***PvC3H69*** | tattgaattcCCTCAAAGAGAGTTCGAGATCG | tataagcttGTTGATGAGGTCCGAGACCCAC |
